# The impact of 3D visual training combined with physical exercise intervention on the visual health of myopic adolescents

**DOI:** 10.1097/MD.0000000000047092

**Published:** 2026-01-09

**Authors:** Zhenhao Yu, Zhuo Chang, Haoxuan Song, Hao Luo

**Affiliations:** a School of Physical Education, Shanxi University, Taiyuan, Shanxi Province, China.

**Keywords:** 3D visual training, myopia, physical exercise, visual health

## Abstract

The prevalence of myopia among adolescents in China has continued to rise in recent years, with an evident trend toward earlier onset and higher degrees of refractive error. This poses significant challenges to learning, daily life, and long-term visual health. Therefore, exploring effective non-pharmacological interventions for adolescent myopia is of practical importance. This study aims to evaluate the effectiveness of 3D visual training combined with physical exercise on visual function and myopia control in adolescents. A total of 188 adolescents with myopia who visited the Optometry Center of our hospital between November 2021 and November 2023 were enrolled and randomly assigned to an observation group (n = 94) or a control group (n = 94). The control group received standard interventions consisting of spectacle correction and eye exercises. The observation group underwent 3D visual training in combination with structured physical exercise. Outcomes assessed before and after intervention included mean uncorrected visual acuity (UCVA) of each eye, overall UCVA, refractive error, axial length, and spherical equivalent. Visual improvement after 6 months and the temporal progression of UCVA within the observation group were also analyzed. Baseline visual and refractive parameters showed no significant differences between the 2 groups (*P* > .05). After the intervention, both groups demonstrated improvements; however, the observation group exhibited significantly greater enhancement in UCVA, accompanied by lower refractive error, shorter axial length, and reduced spherical equivalent compared with the control group (*P* < .05). After 6 months, visual acuity improved in 64 participants (68.09%) in the observation group versus 39 participants (41.49%) in the control group, representing a statistically significant difference (*P* < .05). 3D visual training combined with physical exercise effectively improves uncorrected visual acuity, reduces refractive error, alleviates myopia progression, and promotes visual recovery in adolescents. This combined approach shows promise as a practical, noninvasive strategy for myopia prevention and management.

## 1. Introduction

In recent years, with the continuous changes in China’s urbanization process and people’s learning and lifestyle habits, the incidence of myopia among Chinese adolescents has been rising year by year, showing a trend towards younger onset and higher degrees.^[1,2]^ Myopia has become a major public health issue, severely impacting the learning, life, and future employment of children and adolescents. Furthermore, the various serious ocular complications associated with moderate to high myopia in middle age, such as complicated cataracts, glaucoma, myopic macular degeneration, and retinal detachment, impose a heavy burden on families and society.^[3,4]^ Therefore, seeking an effective method to prevent and treat adolescent myopia is of great practical significance.

Currently, the main methods for treating myopia include drug therapy, health-care measures, corrective glasses therapy, and surgical treatment. These approaches mainly target people who have already developed myopia. They involve relatively high medical costs and only address the symptoms rather than the root cause. Meanwhile, the impacts of contraindications, indications, and complications cannot be ignored.^[5,6]^ 3D vision training is a new method for preventing and treating myopia. Recent studies have found that through a professional large-scale stereoscopic movie playback mode, it trains the 6 extraocular muscles and the ciliary muscle inside the eye. The special patterns stimulate the retina to enhance the health index of the eyeball, achieving the goal of vision training and adjustment.^[7,8]^ In addition, participating in sports such as table tennis and badminton is also considered an effective way to improve eyesight and prevent myopia. On the basis of improving overall physical fitness, these sports can stimulate the recovery of eye-muscle fatigue, exercise the flexibility of the eyeball, and promote the development of the eyes.^[9,10]^

Based on this, this study conducts a retrospective analysis of the clinical data of adolescent myopia patients to further explore the application value of 3D vision training combined with sports in improving adolescent myopia. It is hoped that this study can provide some references for adolescent myopia patients.

## 2. Materials and methods

### 2.1. General information

This study was approved by the Ethics Committee of Shanxi University. A total of 188 myopia patients aged 6 to 17 years who visited the Optometry Center of our hospital from November 2021 to November 2023 were selected. They were divided into an observation group and a control group according to the random number table method, with 94 cases in each group. Before treatment, all the selected subjects underwent routine ophthalmic examinations, including visual acuity tests (both uncorrected and best-corrected visual acuity) using the international standard visual acuity chart, followed by examinations of eye position, eye movement, refractive media, fundus, and fixation characteristics. Those with organic eye diseases were excluded. There were no statistically significant differences in gender, age, visual acuity, and diopter (D) between the 2 groups (*P* > .05), indicating comparability (see Table 1).

**Table 1:** Comparison of general characteristics between groups (n, %; *x̄* ± *s*).

	Control group (n = 94)	Observation group (n = 94)	*t/*χ^*2*^	*P*
Age (yr)	11.34 ± 2.89	11.25 ± 3.01	0.209	.835
Gender				
Male	45	47	0.086	.770
Female	49	47	<0.01	<.01
Visual acuity	0.36 ± 0.15	0.34 ± 0.17	0.855	.394
Refractive error (D)	0.56 ± 0.25	0.53 ± 0.22	0.873	.384

### 2.2. Inclusion and exclusion criteria

Inclusion criteria: Age 6 to 17 years. Patients with mild to moderate myopia, spherical equivalent (SE) between −1.00 and −6.00 D. Consent from the child and parent/guardian, and willingness to cooperate with the training protocol. Interest in sports, enjoyment of physical activity, no language barriers, and the ability to cooperate with the experimenters.

Exclusion criteria: Presence of organic fundus diseases or systemic diseases affecting participation. History of ocular surgery, family history of conditions such as hypertension, heart disease, parental high myopia, or other histories unsuitable for exercise.

### 2.3. Intervention methods

Control group: received intervention with glasses combined with eye exercises. Patients were guided on scientific eye use, integrating patterns such as intermittent eye use, wearing glasses for study, and green reading/writing training into daily life. They were instructed to maintain correct reading and writing postures, limit continuous reading/TV watching to 40 to 45 minutes followed by 10 to 15 minutes of rest, position reading materials to the right of the light source (if using a single source), and use a lampshade to avoid glare. They were required to persist with eye exercises.^[11]^ Specific exercises included: rubbing the upper orbital edge (Tianying point), squeezing and pressing the nose root (Jingming point), and scraping the orbit around the Taiyang point (stimulating Taiyang, Zanzhu, Yuyao, Sizhukong, Chengqi, Tongziliao points). After each exercise, they were to look into the distance to relax their eyes completely.

Observation group: received 3D visual training combined with physical exercise. Visual training was conducted first: patients sat 2 m away from a 3D training screen, following the moving cursor with their eyes as it flashed up, down, left, and right. Initially, the speed was slow, gradually increasing after 2 minutes. Then, based on the patient’s age and degree of myopia, different training films were selected: preschool children chose entertaining films like butterfly flights or animal parks; mentally mature or older patients chose scientifically rigorous, professional films like Star Wars.^[12,13]^ Two films were shown in each session, lasting about 10 minutes total. Patients with low myopia chose low or moderate intensity training. Those with high myopia or longer duration started with low intensity and gradually increased. The training frequency was 3 times/wk, every other day, 12 sessions/mo, 30 min/session, with 3 months constituting 1 course. Visual acuity was checked at least 4 to 6 times per course. Based on the child’s visual acuity after each course, a decision was made regarding the next course. Patients with concomitant amblyopia or strabismus received additional treatments like red light retinal stimulation or peripheral field development as prescribed. Physical exercise (table tennis or badminton) was performed either at home or school, during morning and evening, for no <30 minutes each time. A training log was maintained. Daily exercise time was recorded, and parents were asked to cooperate in supervising completion. Logs were collected and checked regularly to improve compliance. Uncorrected visual acuity (UCVA) and refractive error were checked at least 4 to 6 times per course to adjust the training plan.

### 2.4. Observation indicators

#### 2.4.1. Comparison of mean UCVA between the 2 groups before and after intervention, and overall mean UCVA

UCVA refers to the visual acuity measured without any optical aids such as glasses or contact lenses. A UCVA of 1.0 (or 20/20) or better is generally considered normal, whereas a value below 1.0 indicates refractive errors or amblyopia. Visual acuity was assessed using a standard illuminated logarithm of the minimum angle of resolution chart at a testing distance of 5 m.

#### 2.4.2. Observation of treatment efficacy in the 2 groups after 6 months

To minimize measurement bias, all visual acuity examinations were performed by dedicated optometrists. Treatment efficacy was defined as follows: an improvement in visual acuity was indicated by a reduction in the SE refraction of −0.50 to −2.00 D; no change was defined as a reduction of <−0.50 D or maintenance of the pretreatment refractive error; and deterioration was defined as an increase in the SE refraction of >−1.00 D.

#### 2.4.3. Progression of visual acuity in the observation group at different time points after myopia treatment

Myopia between −1.00 and −3.00 D was considered mild; between −3.00 and −6.00 D moderate; ≥−6.0 D high myopia.

#### 2.4.4. Refractive error and axial length before and after intervention

Refractive examination: cycloplegia using tropicamide, followed by autorefraction using the “Topcon KR-8800” auto-refractometer. Astigmatism was calculated as SE. Axial length measurement: Using the “Kanghua CAS-2000C” ophthalmic ultrasound diagnostic system.

#### 2.4.5. Spherical equivalent (SE) before and after intervention

SE converts astigmatism into a sphere with a similar optical effect. It is calculated by adding half of the cylindrical power to the spherical power: SE = spherical power + 1/2 cylindrical power.^[14,15]^ SE between −0.25 and +0.25 D suggests emmetropia; SE ≤ −0.75 D suggests low myopia; SE between −3.00 and −6.00 D suggests moderate myopia; SE > −6.00 D suggests high myopia.

### 2.5. Statistical analysis

Statistical analysis was performed using Statistical Package for the Social Sciences (SPSS 20.0; SPSS Inc., Chicago). Measurement data are expressed as mean ± standard deviation (*x̄* ± *s*). Count data are expressed as percentages. Single-factor analysis used the chi-square test. Inter-group comparisons used an independent samples *t*-test. *P* value < .05 was considered statistically significant.

## 3. Results

### 3.1. Comparison of mean UCVA per eye before and after intervention

The results showed no significant difference in the mean visual acuity of the left and right eyes between the 2 groups before intervention (*P* > .05). After the intervention, both groups showed improvement compared to pre-intervention, and the observation group’s improvement was significantly greater than that of the control group, with a statistically significant difference (*P* < .05; see Table 2).

**Table 2:** Comparison of mean uncorrected visual acuity per eye between groups before and after intervention (*x̄* ± *s*).

	n	Left eye		Right eye	
		Pre-intervention	Post-intervention	Pre-intervention	Post-intervention
Control	94	0.33 ± 0.16	0.37 ± 0.15	0.32 ± 0.17	0.36 ± 0.16
Observation	94	0.32 ± 0.17	0.54 ± 0.29	0.33 ± 0.15	0.56 ± 0.31
*t*	–	0.415	6.809	0.428	5.558
*P*	–	.678	<.01	.669	<.01

### 3.2. Comparison of overall mean UCVA before and after intervention

Similarly, the results showed no significant difference in the overall mean UCVA between the 2 groups before intervention (*P* > .05). After the intervention, both groups showed improvement compared to pre-intervention, and the observation group’s improvement was significantly greater than that of the control group, with a statistically significant difference (*P* < .05; see Table 3).

**Table 3:** Comparison of overall mean uncorrected visual acuity between groups before and after intervention (*x̄* ± *s*).

	n	Pre-intervention	Post-intervention
Control	94	0.30 ± 0.18	0.38 ± 0.19
Observation	94	0.31 ± 0.16	0.55 ± 0.17
*t*	–	0.403	6.465
*P*	–	.688	<.01

### 3.3. Observation of visual outcomes 6 months after treatment

After 6 months of intervention, visual acuity improved in 64 cases (68.09%) in the observation group, remained unchanged in 24 cases (25.53%), and deteriorated in 6 cases (6.38%). In the control group, visual acuity improved in 39 cases (41.49%), remained unchanged in 30 cases (31.91%), and deteriorated in 25 cases (26.6%). The difference in visual change between the 2 groups was statistically significant (*P* < .05; see Table 4).

**Table 4:** Observation of visual outcomes 6 months after treatment (n, %).

	Improvement	No change	Deterioration	χ^*2*^	*P*
Control (n = 94)	39 (41.49)	30 (31.91)	25 (26.60)	18.380	<.01
Observation (n = 94)	64 (68.09)	24 (25.53)	6 (6.38)		

### 3.4. Comparison of visual acuity progression in the observation group at different time points after myopia treatment

Further research found that after intervention for different durations, visual acuity improvement was significant in the observation group for mild, moderate, and high myopia. Specifically, after 6 months of intervention, improvement was seen in 23 cases (35.93%) with mild myopia, 25 cases (39.06%) with moderate myopia, and 16 cases (25%) with high myopia. This indicates that 3D visual training combined with physical exercise can effectively improve visual acuity, with a more pronounced effect on moderate myopia (see Table 5).

**Table 5:** Comparison of visual acuity progression in the observation group at different time points after myopia treatment (n, %).

Myopia degree	n	Improvement			Total improved	No change	Deterioration
		1 mo	3 mo	6 mo			
Mild	35	3 (13.04)	7 (30.43)	13 (56.52)	23 (35.93)	11 (45.83)	1 (16.67)
Moderate	32	5 (20.00)	9 (36.00)	11 (44.00)	25 (39.06)	4 (16.67)	3 (50.00)
High	27	3 (18.75)	5 (31.25)	8 (50.00)	16 (25.00)	9 (37.5)	2 (33.33)

### 3.5. Comparison of refractive error and axial length before and after intervention

Before the intervention, there were no significant differences between the 2 groups in refractive error or axial length (*P* > .05). After the intervention, both parameters were lower than pre-intervention levels in both groups. Furthermore, the observation group had significantly lower refractive error and axial length compared to the control group, with statistically significant differences (*P* < .05). This indicates that 3D visual training combined with physical exercise can effectively improve refractive error and axial length in myopic individuals, thereby enhancing visual acuity (see Table 6).

**Table 6:** Comparison of refractive error and axial length between groups before and after intervention (*x̄* ± *s*).

	n	Refractive error (D)		Axial length (mm)	
		Pre-intervention	Post-intervention	Pre-intervention	Post-intervention
Control	94	0.51 ± 0.24	0.36 ± 0.15	23.76 ± 0.76	19.55 ± 0.72
Observation	94	0.53 ± 0.17	0.28 ± 0.14	23.59 ± 0.81	15.67 ± 0.68
*t*	–	0.659	3.780	1.484	37.984
*P*	–	.511	<.01	.140	<.01

### 3.6. Comparison of spherical equivalent before and after intervention

The results showed no significant difference in SE between the 2 groups before the intervention. After the intervention, the SE in the observation group was lower than both its pre-intervention level and that of the control group, with a statistically significant difference (*P* < .05). This indicates that 3D visual training combined with physical exercise can effectively reduce the SE in myopic individuals, alleviating issues related to lag of accommodation (see Table 7).

**Table 7:** Comparison of spherical equivalent between groups before and after intervention (*x̄* ± *s*).

	n	Pre-intervention	Post-intervention
Control	94	−0.78 ± 0.23	−0.68 ± 0.17
Observation	94	−0.76 ± 0.21	−0.57 ± 0.16
*t*	–	0.623	4.568
*P*	–	.534	<.01

## 4. Discussion

Adolescent myopia is often caused by factors such as dim lighting in the learning environment and poor reading or writing postures.^[16]^ With technological advancements, there are increasingly more methods for myopia correction, broadly divided into surgical and non-surgical categories. Surgery primarily involves laser procedures, while non-surgical methods mainly include wearing spectacles or orthokeratology lenses to correct vision. Additionally, eye exercises and fogging therapy are widely used. However, as most adolescents find it difficult to adhere to these methods or perform them correctly, the effectiveness is often limited.^[17,18]^ Therefore, choosing a convenient and effective treatment method is crucial.

Research has confirmed that the absolute and relative accommodative amplitudes of myopic eyes are lower than those of emmetropic eyes; the essence of myopia is a weakening of accommodative power. Myopic eyes often experience asthenopia due to abnormal vergence and accommodation functions.^[19]^ Based on the accommodation theory of myopia, this study employed a combined intervention of 3D visual training and physical exercise to improve accommodative ability. Visual training was administered first. The study found that post-intervention UCVA (for both individual eyes and overall) was better than pre-intervention levels in both groups, and the observation group outperformed the control group. This is primarily because this training regulates functions like fusion training and dynamic stereoscopic vision training, enhancing the eye’s ability to focus at near and far distances. It trains binocular convergence and divergence, the eye’s ability to converge, and ocular motility, including saccades, pursuits, and tracking, making the patient’s eye movements more flexible.^[20]^

In addition, this study also showed that after 6 months of intervention, 64 cases in the observation group had a visual improvement, for 68.09%, and 39 cases in the control group had a visual improvement, accounting for 41.49%, which was significant. Further research found that after different time of intervention, the observation group had a significant improvement in visual acuity of mild, moderate and high myopia. This shows that the combined intervention of 3D visual training and sports training can effectively control the development of myopia in adolescents. With the passage of time, the progression of vision in the observation group gradually slowed from 1, 3, and 6 months, and the vision improved slowly. The reason is mainly that the 3D training is an active myopia treatment method. Applies advanced multimedia high-tech technology to strengthen eye protection in the game. The form is novel and targeted. Teenagers have changed from forced treatment to active participation in the past. The compliance is better.^[21,22]^ At the same time, when playing table tennis or badminton, both eyes must stare at the ball. The moves back and forth in the air quickly, sometimes far and sometimes near, rotating and changing. This makes the ciliary muscle contract and relax continuously, so that the ciliary muscle exercises fully and rests, eliminating eye fatigue and improving or restoring the automatic regulation ability of the eyes, playing a role in preventing and treating myopia.^[23,24]^

The cornea, aqueous humor, lens, and vitreous body together constitute the eye’s refractive system.^[25]^ Our results show that post-intervention refractive error and axial length were lower than pre-intervention levels in both groups, and the observation group had significantly lower values than the control group. The lens, located behind the iris, is a biconvex lens. Normally, through the contraction and relaxation of the ciliary muscle, the lens thickness increases or decreases, allowing the image focus to fall precisely on the retina, forming a clear image.^[26,27]^ Adolescent myopia often occurs due to improper eye use, insufficient accommodation, or excessive accommodation. Training accommodative power essentially involves training the extraocular muscles and the ciliary muscle function. Three-dimensional visual training actively addresses this by requiring effort to focus on a specific point in the image and trying to see the image as far away as possible, training the brain’s control over the ciliary muscle. When the image moves, relaxed viewing of the full scene provides training for ciliary muscle movement. Repeating this process can improve the amplitude of accommodation of the ciliary muscle.^[28,29]^ Physical exercise, guided by professional instructors, can achieve optimal condition and function.

Meanwhile, with advances in medical understanding, visual training is believed to help maintain a stable blood supply and can effectively increase the speed, amplitude, and force of muscle movement after training, improving the amplitude of accommodation and symptoms of accommodative lag.^[30-32]^ Our results confirm this: patients in the observation group who received the standardized combined intervention of 3D visual training and physical exercise showed a significant reduction in SE post-intervention, indicating that this method can effectively control the myopia progression process and alleviate issues of accommodative lag. This study has several limitations. First, the follow-up period was relatively short, and therefore the long-term sustainability of the observed visual improvements remains uncertain. Future studies should extend the follow-up duration to evaluate whether the benefits of 3D visual training combined with physical exercise can be maintained over time. Second, lifestyle factors known to influence myopia progression – such as near-work time, outdoor activity duration, screen exposure, and sleep quality – were not systematically assessed or controlled in our study. These uncontrolled variables may have affected individual visual outcomes. Further research incorporating detailed lifestyle assessments and controlling for these factors is needed to better clarify the independent effect of the intervention.

In summary, 3D visual training combined with physical exercise can effectively improve visual acuity in adolescents, slow the progression of myopia, promote visual recovery, and ameliorate accommodative lag. It is worthy of clinical promotion and application. However, due to the limitation of sample size, its preventive and therapeutic effects on myopia require further in-depth study. Subsequent research will appropriately increase the sample size and intervention period to better observe its clinical efficacy and application value.

## Author contributions

**Conceptualization:** Zhenhao Yu, Zhuo Chang, Haoxuan Song, Hao Luo.

**Data curation:** Zhenhao Yu, Zhuo Chang, Haoxuan Song, Hao Luo.

**Formal analysis:** Zhenhao Yu, Zhuo Chang, Haoxuan Song, Hao Luo.

**Funding acquisition:** Zhuo Chang, Hao Luo.

**Investigation:** Hao Luo.

**Writing** – **original draft:** Zhenhao Yu, Zhuo Chang, Haoxuan Song, Hao Luo.

**Writing** – **review & editing:** Zhenhao Yu, Zhuo Chang, Haoxuan Song, Hao Luo.
